# Search for Antimicrobial Activity Among Fifty-Two Natural and Synthetic Compounds Identifies Anthraquinone and Polyacetylene Classes That Inhibit *Mycobacterium tuberculosis*

**DOI:** 10.3389/fmicb.2020.622629

**Published:** 2021-01-18

**Authors:** Luiz A. E. Pollo, Erlon F. Martin, Vanessa R. Machado, Daire Cantillon, Leticia Muraro Wildner, Maria Luiza Bazzo, Simon J. Waddell, Maique W. Biavatti, Louis P. Sandjo

**Affiliations:** ^1^Programa de Pós-Graduação em Farmácia, CCS, Universidade Federal de Santa Catarina, Florianópolis, Brazil; ^2^Department of Global Health and Infection, Brighton and Sussex Medical School, University of Sussex, Brighton, United Kingdom; ^3^Programa de Pós-Graduação em Química, CFM, Departamento de Química, Universidade Federal de Santa Catarina, Florianópolis, Brazil

**Keywords:** *Mycobacterium tuberculosis*, drug discovery, natural product, synthetic polyacetylenes, antimicrobial drug resistance

## Abstract

Drug-resistant tuberculosis threatens to undermine global control programs by limiting treatment options. New antimicrobial drugs are required, derived from new chemical classes. Natural products offer extensive chemical diversity and inspiration for synthetic chemistry. Here, we isolate, synthesize and test a library of 52 natural and synthetic compounds for activity against *Mycobacterium tuberculosis*. We identify seven compounds as antimycobacterial, including the natural products isobavachalcone and isoneorautenol, and a synthetic chromene. The plant-derived secondary metabolite damnacanthal was the most active compound with the lowest minimum inhibitory concentration of 13.07 μg/mL and a favorable selectivity index value. Three synthetic polyacetylene compounds demonstrated antimycobacterial activity, with the lowest MIC of 17.88 μg/mL. These results suggest new avenues for drug discovery, expanding antimicrobial compound chemistries to novel anthraquinone and polyacetylene scaffolds in the search for new drugs to treat drug-resistant bacterial diseases.

## Introduction

Tuberculosis (TB) is an infectious disease that is among the top 10 causes of death worldwide, and the leading bacterial cause of death. In 2019, an estimated 10 million people developed TB and 1.4 million people died as result of the disease (World Health Organization, 2020). TB treatment requires the use of multiple drugs for at least 6 months. This lengthy therapy together with adverse drug reactions contribute to patient non-adherence, resulting in treatment failure and the development of drug-resistant *Mycobacterium tuberculosis*. The emergence of multidrug-resistant (MDR) TB and extensively drug resistant (XDR) TB is undermining global control efforts. In 2019, 3.3% of new TB cases and 17.7% of previously treated cases were rifampicin-resistant (RR)/MDR-TB. There were an estimated 465,000 incident cases of RR-TB in the same year, of which 78% were MDR-TB (World Health Organization, 2020). Therefore, there is an urgent need, recognized by the World Health Organization (World Health Organization, 2014), for new drugs to treat drug-resistant TB, and to shorten therapy for drug-sensitive TB. However, despite sustained international efforts, only pretomanid, delamanid and bedaquiline have been marketed as new drugs for TB treatment in the last 40 years (The Working Group for New TB Drugs, 2020). Intensified research and innovation are needed to meet the End TB Strategy targets set for 2030, a key priority of which is to discover new drugs based on new chemical entities (World Health Organization, 2014).

Over the past 60,000 years, plant-derived medicines have been used as decoctions, infusions and tinctures to improve human health (Solecki and Shanidar, 1975). Numerous studies have attempted to correlate ethnological knowledge with the scientific evidence base (De Smet, 1997). Natural products are an essential source of biologically active components (Thomford et al., 2018; Wright, 2019), and naturally occurring secondary metabolites have inspired the development of therapeutic drugs for infectious, cardiovascular, and degenerative diseases (Thomford et al., 2018; Lautié et al., 2020). Natural products are composed of numerous structural diversities, often containing complex hydrocarbon skeletons that have been explored to produce libraries of biologically relevant derivatives (Salomon and Schmidt, 2012; Pascolutti and Quinn, 2014). *Dorstenia* plant species are used in sub-Saharan African and South American countries as herbal medicines to treat cough, pneumonia and other infectious diseases such as malaria, syphilis, and hepatitis (Togola et al., 2008; Teklehaymanot, 2009; Bieski et al., 2012; Adem et al., 2018). Prenylated flavonoids obtained from *Dorstenia* species showed antibacterial activity against a broad-spectrum of bacteria, including *M. tuberculosis* (Mbaveng et al., 2012). Secondary metabolites from *Erythrina senegalensis*, the Senegal coral tree, have been demonstrated to exhibit strong inhibition of methicillin resistant *Staphylococcus aureus*, *Enterococcus faecalis*, and *Bacillus subtilis* (Koné et al., 2004). Damnacanthal, an anthraquinone obtained from *Pentas schimperi*, displayed moderate activity against the *Trypanosoma cruzi* amastigote (Sandjo et al., 2016b). As numerous biologically active molecules have been inspired by the organic constituents of plants, our group prepared a series of pyridine and chromene derivatives (Pollo et al., 2017; Martin et al., 2018). These compounds were evaluated for their antiparasitic activities against *Leishmania amazonensis* and *T. cruzi* amastigotes. Three pyridines and three chromenes inhibited *T. cruzi* with IC_50_ values less than 7 μM. Similarly, a coumarin scaffold was used to generate anti-TB agents, while polyacetylenes from plants and pyridine derivatives have been shown to express antimycobacterial activities (Rogoza et al., 2010; Somagond et al., 2019; Kumar et al., 2020).

Here, we report the extraction, synthesis, and anti-*M. tuberculosis* activity of 52 compounds: six plant secondary metabolites from *Dorstenia kameruniana*, *Dorstenia mannii*, *P. schimperi*, and *E. senegalensis*, and 46 synthetic compounds including 20 dihydropyridines, 12 pyranocoumarins, seven chromenes, one oxazinone, one conjugated ester and five polyacetylenes. This study identifies anthraquinone and polyacetylene compounds as the basis for novel drug discovery toward new therapeutic options for drug-resistant TB.

## Materials and Methods

### Origin of the Natural Products

**FIGURE 1:**
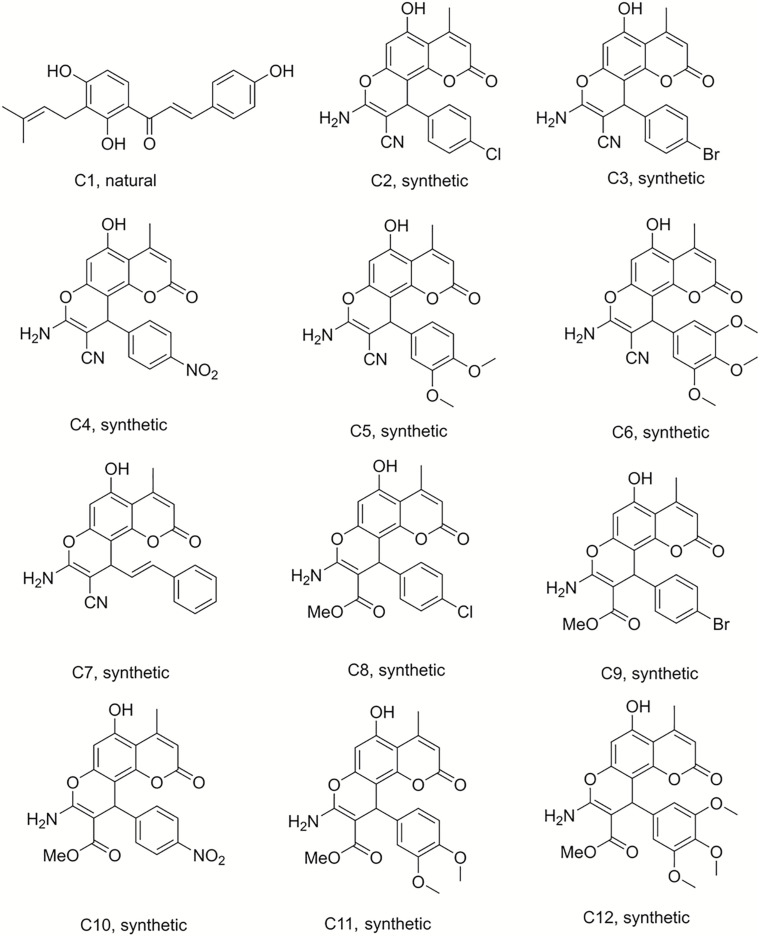
Structures of the compounds tested for anti-*M. tuberculosis* activity: Chalcone C1, and the pyranocoumarins C2–C12 (Abegaz et al., 1998; Martin et al., 2018).

**FIGURE 2:**
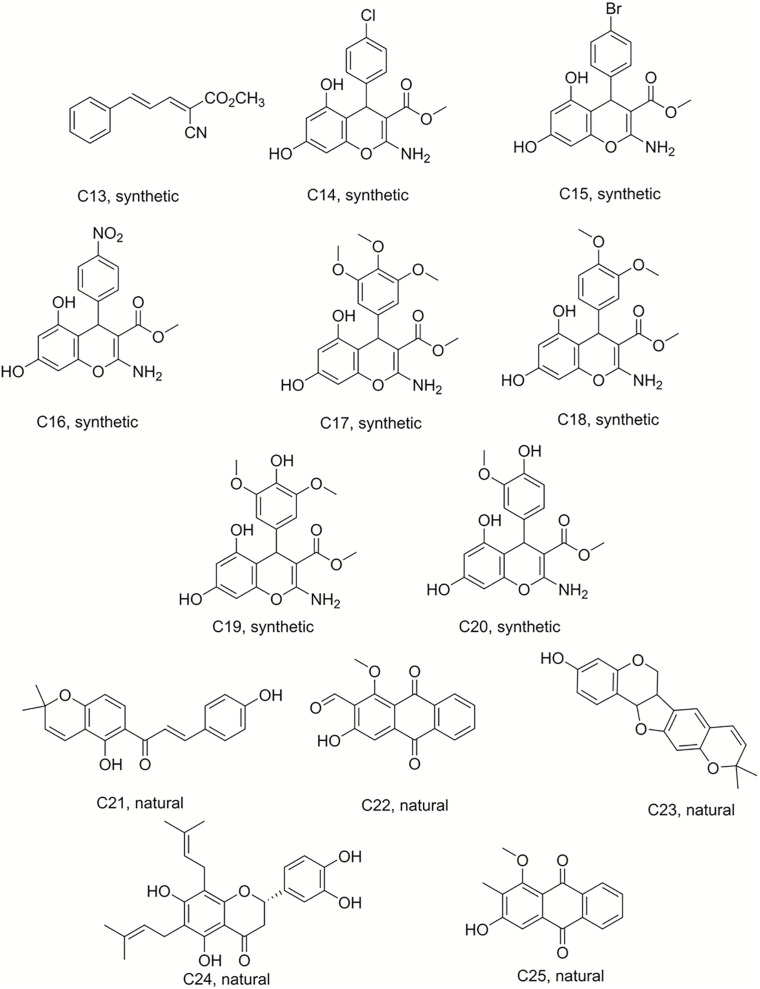
Structures of the compounds tested for anti*-M. tuberculosis* activity: Ester C13, chromenes C14–C20, and natural products C21–C25 (Ngadjui et al., 1998; Kuete et al., 2014, 2015a; Martin et al., 2018).

Isobavachalcone (C1) was isolated from *D. kameruniana*, while 4-hydroxylonchocarpine (C21) and 6,8-diprenyleriodictyol (C24) were obtained from *D. mannii* (Abegaz et al., 1998; Ngadjui et al., 1998). Damnacanthal (C22) and its reduced derivative (C25) were isolated from *P. schimperi* (Kuete et al., 2015a). Isoneorautenol (C23) was extracted from *E. senegalensis* (Kuete et al., 2014). The structures of these natural secondary metabolites are displayed in Figures 1, 2.

### Preparation of the Pyranocoumarin Derivatives

Compound 50 (C50) was synthesized by treating phloroglucinol at 60°C for 6 h with one equivalent of ethyl acetoacetate and a catalytic amount of polyphosphoric acid. This coumarin was then used as the starting material to prepare a series of pyranocoumarins. C50, arylaldehydes, malononitrile, and K_2_CO_3_ were submitted to reflux conditions to generate compounds C2–C7. Compounds C8–C12 were obtained using the same one-pot conditions except that malononitrile was replaced by methyl α-cyanoacetate. C13 was a by-product formed from the Knoevenagel condensation reaction of cinnamaldehyde and α-cyanoacetate in the same reaction conditions. The structures are detailed in Figures 1, 2, 4 (Martin et al., 2018).

### Preparation of Chromenes

Chromene derivatives C14–C20 were obtained by a direct reaction of phloroglucinol, arylaldehydes and methyl α-cyanoacetate in alkaline reflux conditions. We have previously described the synthesis and the identification of C2–C20 (Martin et al., 2018), shown in Figure 2.

### Preparation of Dihydropyridine Derivatives and Analogs

**FIGURE 3:**
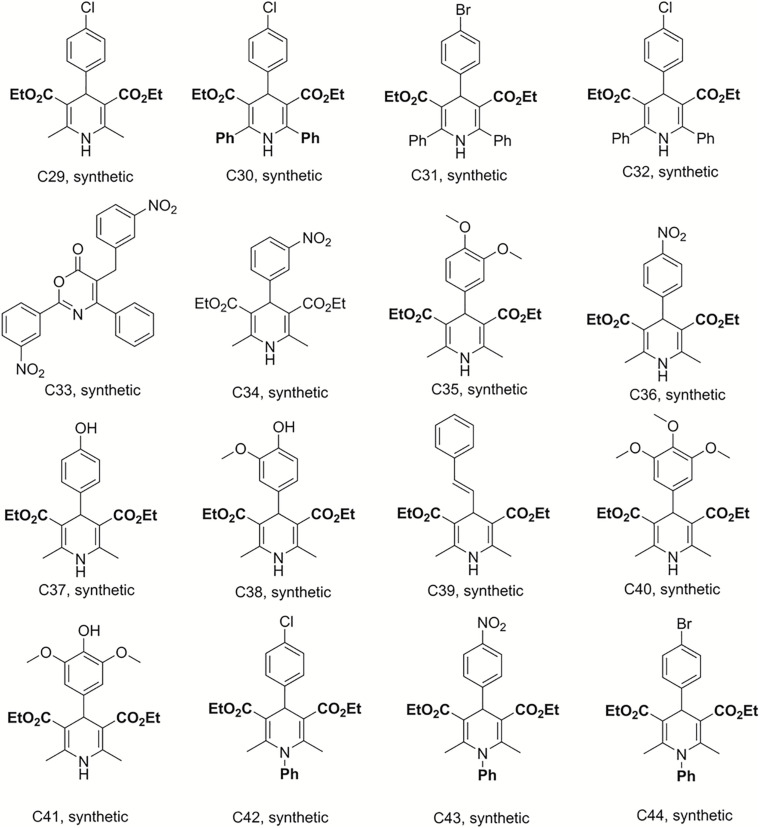
Structures of the compounds tested for anti*-M. tuberculosis* activity: Dihydropyridines C29–C44 (Sandjo et al., 2016a; Pollo et al., 2017).

**FIGURE 4:**
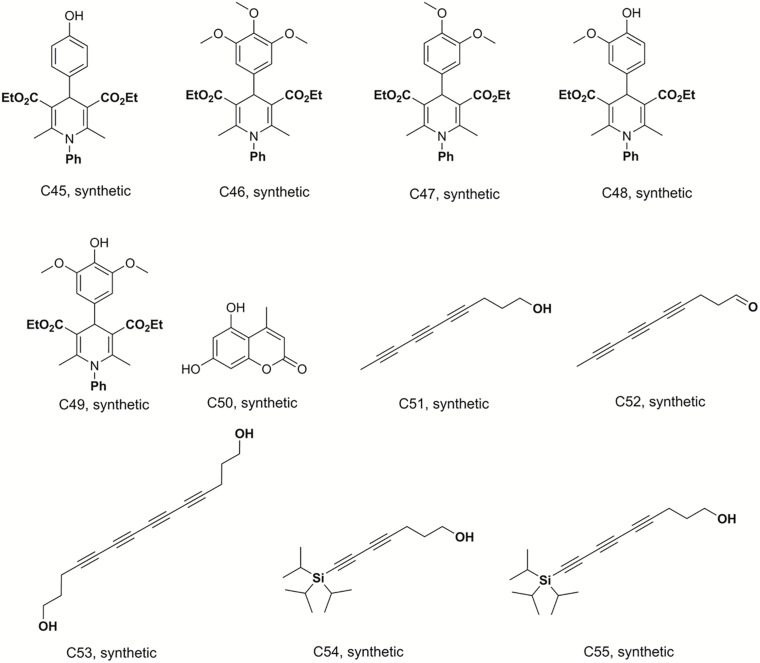
Structures of the compounds tested for anti*-M. tuberculosis* activity: Dihydropyridines C45–C49, coumarin C50, and polyacetylenes C51–C55 (Sandjo et al., 2016a; Pollo et al., 2017; Machado et al., 2018; Martin et al., 2018).

Bismuth chloride was used to promote the reaction, which was carried out in tetrahydrofuran under reflux conditions and was stirred for 6 h to synthesize compounds C29–C33 (Sandjo et al., 2016a). C34–C41 were obtained from the same reaction conditions replacing ethyl benzoylacetate with ethyl acetylacetate in reflux and free catalyst conditions to prepare these dihydropyridine analogs. C42–C49 were also synthesized in reflux conditions without any catalyst, replacing ammonium acetate with aniline. The preparation of these dihydropyridine analogs has been described (Pollo et al., 2017), and their structures are displayed in Figures 3, 4.

### Preparation of the Polyacetylene Derivatives

Deca-4,6,8-triyn-1-ol (C51), deca-4,6,8-triynal (C52), 7-(triisopropylsilyl)hepta-4,6-diyn-1-ol (C54) and 9-(triisopropylsilyl)nona-4, 6-,8-triyn-1-ol (C55) were previously synthesized, identified and reported by Machado and co-workers (Machado et al., 2018). Tetradeca-4,6,8,10-tetrayne-1,14-diol (C53) was prepared by homodimerization reaction in a 25 mL, two neck, round-bottom flask equipped with rubber septum and a magnetic stir bar, filled with a solution of hepta-4,6-diyn-1-ol (0.1 g; 0.925 mmol; 1 equiv.) in CH_3_CN (10 mL). To this solution was added Cu(OAc)_2_ (0.353 g; 1.94 mmol; 2.1 equiv) and K_2_CO_3_ (0.153 g; 1.11 mmol; 1.2 equiv. The resulting mixture was stirred vigorously at room temperature overnight (∼18 h). The organic extract was washed with brine (20 mL), dried over MgSO_4_, filtered, and concentrated under reduced pressure. Purification by column chromatography on silica gel (elution with 40% EtOAc-hexanes) afforded 0.063 g (32 %) of tetradeca-4,6,8,10-tetrayne-1,14-diol as a pale yellow oil. mp 116.5 - 117°C; *R.f*: 0.75 (40% hexane-ethyl acetate; ^1^H NMR (300 MHz, CD_3_OD) δ in ppm: 3.61(t, 4H), 2.44 (t, *J* = 7.15 Hz, 4H), 1.74 (m, 4H). ^13^C-NMR (75 MHz, CD_3_OD) δ in ppm: 81.4; 66.1; 62.1; 61.2; 60.8; 31.9; 16.5. The structures of these compounds are shown in Figure 4.

### Bacteria and Culture Conditions

*Mycobacterium tuberculosis* H37Rv reference strain was cultured in Middlebrook 7H9 broth (Sigma-Aldrich) supplemented with albumin dextrose catalase (ADC, 10% v/v) and Tween 80 (0.05% v/v) at 37°C. Optical density was measured using a spectrophotometer (Promega) at absorbance 600 nm. Colony forming units (CFU) were determined by serially diluting cultures onto Middlebrook 7H10 agar (Sigma-Aldrich) supplemented with 0.5% glycerol and oleic acid albumin dextrose catalase (OADC, 10% v/v) and incubated at 37°C for 4 weeks. All *M. tuberculosis* work was conducted in containment level three laboratories, following institutional biosafety and biosecurity standards for working with hazard group three pathogens.

### Antimycobacterial Activity

All compounds were prepared as 10 mg/mL stock solutions in sterile dimethyl sulfoxide (DMSO), except for rifampicin which was prepared with 90% w/v methanol. Single use aliquots of compounds were prepared and stored at −20°C. Log phase *M. tuberculosis* cultures were adjusted from 2 × 10^5^ to 5 × 10^5^ CFU/mL, added to 96 well microtiter plates containing test compounds at a final concentration of 100 μg/mL (1% DMSO final concentration), and incubated for 7 days at 37°C. To determine cell viability, CellTiter-Blue (Promega) was added to the plates at a final concentration of 10% v/v and incubated overnight (Franzblau et al., 1998). Fluorescence was measured at excitation 580–640 nm and emission 520 nm using a Glomax Discover plate reader (Promega). Hits were classified as any compound that inhibited growth by ≥40% compared to drug-free *M. tuberculosis* controls. This cut-off was selected to capture the range of antimicrobial activity of related chemical compounds, rather than highlight individual compounds with superior activity. Minimum inhibitory concentrations (MIC) of hit compounds were determined using a resazurin microtiter plate assay (REMA) with CellTiter-Blue (Promega) as described above, with two-fold serial dilutions from 100 μg/mL to 1.56 μg/mL. The MIC experiments were repeated in triplicate. MICs were calculated by non-linear regression, fitting these data to a modified Gompertz equation for MIC determination, using GraphPad Prism 8. Validation of this assay for established TB drugs (isoniazid and linezolid) is detailed in Supplementary Figure 1.

### Cytotoxicity Assay

The human monocytic THP-1 cell line was maintained at 37°C, 5% CO_2_ in supplemented RPMI 1640 medium (Gibco, Life Technologies) containing 2 mM L-glutamine (Gibco, Life Technologies) and 10% heat inactivated FBS (Pan Biotech). The cells were passaged every 4 days. To measure compound cytotoxicity, 5 × 10^4^ monocytes per well were added to 96 well plates and treated for 24 h with the compounds at concentrations ranging from 200 μg/mL to 1.56 μg/mL. Cell viability was determined by fluorescence quantification after 2 h incubation with CellTiter-Blue (Promega), according to the manufacturer’s instructions. Fluorescence was measured at excitation 580–640 nm and emission 520 nm using a Glomax Discover plate reader (Promega). Fluorescence values were adjusted for media fluorescence and the inhibitory concentration 50% (IC_50_) was calculated using Graphpad Prism 8. The selectivity index (SI) of each compound was calculated by dividing the IC_50_ by the corresponding *M. tuberculosis* MIC value.

## Results and Discussion

The discovery of biologically active new chemical entities is crucial to developing novel chemotherapeutic agents against drug resistant bacterial infections, including TB. Chemistry uses synthetic approaches and analytic techniques to identify and isolate natural products and to produce small molecules bearing diverse hydrocarbon skeletons for preclinical studies (Lombardino and Lowe, 2004; Campbell et al., 2018). To contribute to the search for new anti-tubercular agents, a library of 52 natural and synthetic compounds (Figures 1–4) were tested against log phase *M. tuberculosis*. The library contained compounds with diverse chemistries including 11 pyranocoumarins (C2–C12), seven chromenes (C14–C20), one conjugated arylester (C13), two chalcones (C1 and C21), two anthraquinones (C22 and C25), one pterocarpan (C23), one flavanone (C24), 20 dihydropyridines (C29–C32 and C34–C49), one oxazinone (C33), one coumarin (C50), and five polyacetylenes (C51–C55).

### Antimycobacterial Activity

**FIGURE 5:**
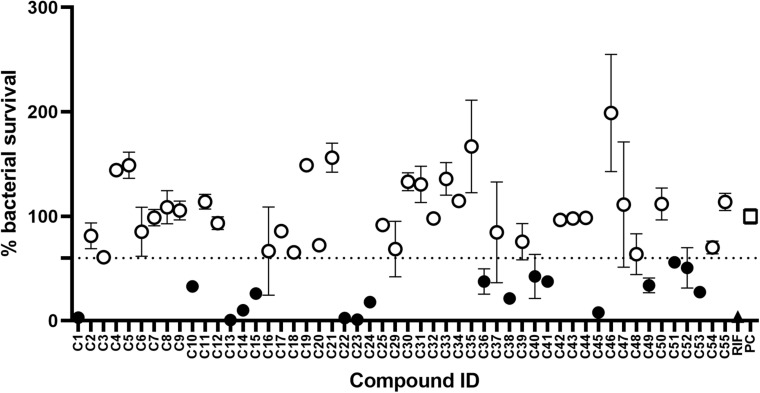
Compound library screen against *Mycobacterium tuberculosis*. Log phase *M. tuberculosis* was treated with compounds at 100 μg/mL for 7 days, bacterial survival was measured after overnight incubation with CellTiter-Blue. Data points are expressed as mean % survival compared to drug-free controls. Error bars represent the standard deviation. Hits were classified as any compound that inhibited growth by ≥40%. Filled circles identify hit compounds. Empty circles indicate compounds not classified as hits. The mean bacterial survival ≤60% shown as a dotted line. Filled triangle (RIF) marks rifampicin-treated bacilli. Empty square (PC) indicates drug-free positive controls.

**FIGURE 6:**
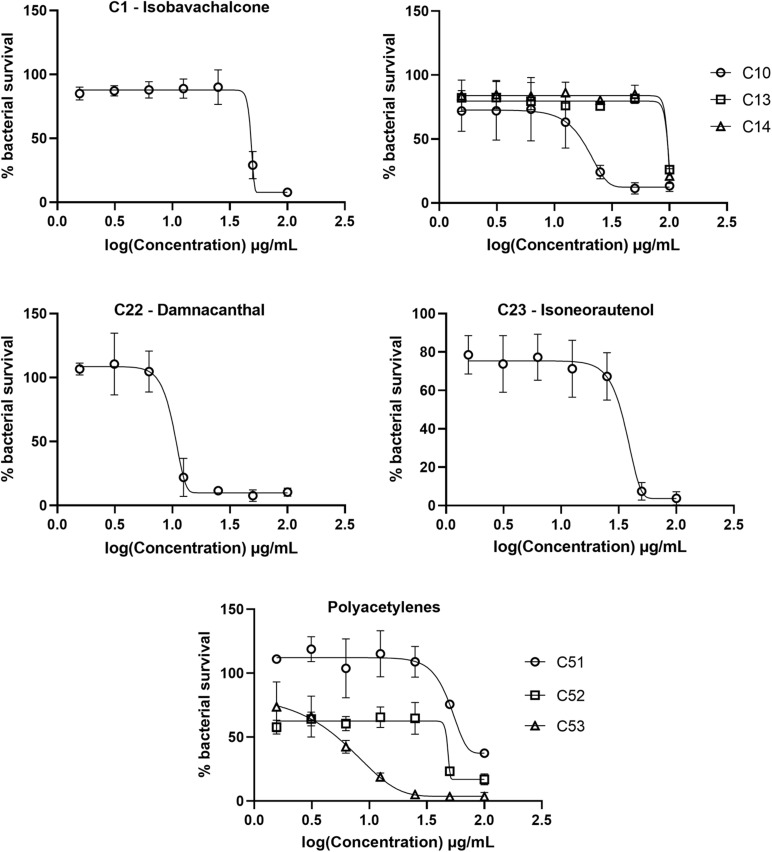
Dose response curves of *Mycobacterium tuberculosis* treated with hit compounds. *M. tuberculosis* was treated for 7 days with compounds ranging in concentration from 100 μg/mL to 1.56 μg/mL. Bacterial survival was measured after overnight incubation with CellTiter-Blue to establish minimum inhibitory concentrations (MICs). Data points are expressed as mean % survival relative to drug-free controls from duplicate biological replicates. Error bars represent the standard deviation.

The initial screen of the compound library at 100 μg/mL, using the colorimetric CellTiter-Blue assay to measure mycobacterial viability, identified 17 of the 52 compounds that inhibited *M. tuberculosis* survival by at least 40% in comparison to drug-free bacilli (Figure 5). Five compounds inhibited *M. tuberculosis* at a level similar to the first line anti-TB drug rifampicin. To verify the results of the initial screen and to establish compound activity, minimum inhibitory concentrations (MICs) were determined for all 17 hits using the microbroth dilution method. Compound C22 (the natural product damnacanthal) displayed the greatest activity, with a MIC of 13.07 μg/mL, followed by the polyacetylene C53 with a MIC of 17.88 μg/mL (Figure 6 and Table 1). Compounds C1, C10, C23, C51, and C52 were demonstrated to have MICs against *M. tuberculosis* between 25 and 71 μg/mL (Figure 6 and Table 1). Compounds C13, C14, C15, C24, C36, C38, C40, C41, C45, and C49 resulted in MICs ≥100 μg/mL. Thus, we identified seven chemically diverse compounds that inhibited *M. tuberculosis*.

**TABLE 1:** Antimycobacterial and cytotoxic activities of hit compounds against *Mycobacterium tuberculosis*.

Compound	MIC (μg/mL)	IC_50_ (μg/mL)	Selectivity index
C1	51.77	45.85	0.89
C10	29.13	64.18	2.20
C13	105.6	95.94	0.91
C14	105.4	47.22	0.45
C15	>100	N/D	N/A
C22	13.07	21.41	1.64
C23	49.22	44.27	0.90
C24	>100	N/D	N/A
C36	>100	N/D	N/A
C38	>100	N/D	N/A
C40	>100	N/D	N/A
C41	>100	N/D	N/A
C45	>100	N/D	N/A
C49	>100	N/D	N/A
C51	70.69	170.5	2.41
C52	50.58	N/D*	N/A
C53	17.88	17.00	0.95

Detailing MIC, IC_50_, and selectivity index values. MIC, minimum inhibitory concentration against *M. tuberculosis*; IC_50_, inhibitory concentration 50% for human monocyte THP-1 cells; N/D, not done; N/A, not applicable. *IC_50_ for C52 was not conducted due to compound degradation over time.

The top hit against *M. tuberculosis* was damnacanthal (C22 – MIC of 13.07 μg/mL), a naturally occurring secondary metabolite isolated from the tropical plant *P. schimperi* (Figure 6), which has previously been demonstrated to have antibacterial activity against *S. aureus* and *Pseudomonas aeruginosa*. Further investigation revealed that its inhibitory effect on *S. aureus* might be related to an increase in toxic reactive oxygen species (Comini et al., 2011). This natural metabolite has also been demonstrated to have prominent antifungal activity against *Aspergillus ochraceus*, *Aspergillus niger* and *Candida lipolytica* (Ali et al., 2000). It also displayed anti-parasitic properties against *T. cruzi* amastigotes (Sandjo et al., 2016b). The antimycobacterial activity of damnacanthal (C22) is likely linked to its aldehyde functional group as compound C25 (rubiadin 1-methyl ether), its reduced form, showed no anti-*M. tuberculosis* activity.

The dimeric polyacetylene C53 (tetradeca-4,6,8,10-tetrayne-1,14-diol) was the second most active compound against *M. tuberculosis*, with a MIC of 17.88 μg/mL. Several naturally occurring polyacetylene alcohols with acyclic hydrocarbon backbones have been reported with moderate to significant antimycobacterial activity against *Mycobacterium fortuitum*, *Mycobacterium avium*, *Mycobacterium aurum*, and *M. tuberculosis* H37Ra (Kobaisy et al., 1997; Schinkovitz et al., 2008; Haoxin et al., 2012). These metabolites also showed a wide spectrum of antibacterial action against Gram positive bacteria (*S. aureus* and *B. subtilis*), Gram negative bacteria (*Escherichia coli* and *P. aeruginosa*), *Candida albicans*, *M. tuberculosis*, and isoniazid-resistant *M. avium* (Kobaisy et al., 1997; Christensen, 2011), supporting the antimicrobial activity presented here using a synthetic polyacetylene. Other synthetic polyacetylenes of the series tested (C51 and C52) were active against *M. tuberculosis* (Figure 6), while C54 and C55 were not. C52 was more active than C51, with MICs of 50.58 μg/mL and 70.69 μg/mL, respectively, and both compounds differ from each other by the hybridization of atoms in the C-O bond. C53, obtained from the homodimerization of C51, showed the highest anti-*M. tuberculosis* activity among the polyacetylenes, suggesting that the observed bioactivity might be promoted by the high conjugated π-electron system.

The natural product isobavachalcone (C1), isolated from *D. kameruniana*, was moderately inhibitory to *M. tuberculosis* with a MIC of 51.77 μg/mL (Figure 6). This compound has been previously determined to be active against *M. tuberculosis* (Chiang et al., 2010), verifying our library screen. Antibacterial activity against both Gram positive and Gram negative bacteria (Mbaveng et al., 2008) has also been observed, alongside antifungal activity, inhibiting *C. albicans* (ElSohly et al., 2001). Prenylated chalcones structurally close to isobavachalcone have also been identified as broad-spectrum antibacterial agents (Sugamoto et al., 2011). Isoneorautenol (C23), a natural secondary metabolite isolated from *E. senegalensis*, had a MIC of 49.22 μg/mL against *M. tuberculosis* (Figure 6), and has also been shown to inhibit growth of the fast-growing non-pathogenic *Mycobacterium smegmatis* and Gram negative bacteria (Mitscher et al., 1988; Mbaveng et al., 2015). Of note, the natural product 4-hydroxylonchocarpine (C21), a flavonoid class chalcone isolated from *D. mannii*, was not mycobactericidal despite previous reports of antibacterial activity (Kuete et al., 2013).

The synthetic chromene C10 showed good antimycobacterial activity with a MIC of 29.13 μg/mL against *M. tuberculosis* (Figure 6). This compound, prepared for a previous study, demonstrated anti-parasitic action against both *T. cruzi* and *L. amazonensis* (Martin et al., 2018). Chromene derivatives bearing trisubstituted amines were previously reported as antimycobacterial against *M. tuberculosis* H37Rv (Raj and Lee, 2020). C10, the most active compound among the pyranocoumarin derivatives, differs from the others with the substituent group on the aryl moiety and the ester function on the pyran ring. C16, different from C10 by the coumarin ring, was not active against *M. tuberculosis*. However, when the NO_2_ group in C16 was replaced by the halogen atom (compound C14), a weak antimycobacterial activity was observed (Figure 6). C13 also contains a high conjugated π-electron system, although not as linear as in the C53 structure, which might support the weak activity of this compound against *M. tuberculosis* (Figure 6).

### Compound Toxicity

Compounds that exhibited significant antimycobacterial activity were assessed for toxicity toward human monocytic (THP-1) cells *in vitro*, and the IC_50_ and selectivity index (SI) values were calculated for each compound. Compounds C1, C13, C14, C23, and C53 exhibited SI values lower than 1, while compounds C10, C22, and C51 exhibited SI values greater than 1, but limited to a maximum of 2.41 (Table 1). Therefore, efforts will be required to improve the selectivity of these compounds for mycobacteria.

Several of these compounds have been previously described to have moderate to low cytotoxicity to healthy cells. During evaluation of isobavachalcone (C1) on intracellular parasites, C1 showed some toxicity to THP-1 cells with a cytotoxic concentration 50% (CC_50_) of 11.65 μg/mL. Compound C22 in the same study inhibited the growth of THP-1 cells with a CC_50_ of 8.87 μg/mL (Sandjo et al., 2016b). Compounds C13 and C14 were also identified to be moderately toxic to human monocyte cells alongside their intracellular antiprotozoal activity (Martin et al., 2018), correlating with our findings here. Isoneorautenol (C23) was reported as antiproliferative without showing cytotoxicity to AML12 hepatocytes at concentrations up to 123.46 μM (Kuete et al., 2014; Kuete et al., 2015b). No cytotoxicity studies have been performed on the polyacetylenes (C51–C53). The intermediate MIC values for these compounds against *M. tuberculosis* (as shown in this study) are close to the IC_50_ values, resulting in greater potential for cytotoxicity. The reduction of this toxicity should be a priority for further compound development.

## Conclusion

New drugs are required to treat drug-resistant TB and the rising threat of antimicrobial drug resistance (AMR). Natural products, and synthetic compounds derived and inspired by natural products, offer an extensive diversity of bioactive chemical structures for drug discovery. Natural product screens offer the opportunity to identify new chemical entities, with likely novel modes of action to overcome existing bacterial drug resistance mechanisms. Here, we report the anti-TB activity of 52 natural and synthetic compounds selected to have different hydrocarbon scaffolds and reported antimicrobial or antiparasitic activities. We identify two compounds (C22 damnacanthal, and the dimeric polyacetylene C53) with MIC values <20 μg/mL against *M. tuberculosis*, and five with MIC values between 25 and 71 μg/mL. The synthetic chromene C10 and polyacetylene C51 with moderate antimycobacterial activity displayed low toxicity compared to the other active compounds. This study suggests that the anthraquinone damnacanthal (C22) and synthetic polyacetylenes (C53, C52, and C51) deserve further chemical investigation as novel antimycobacterial scaffolds, and biological experimentation to elucidate mechanism of action in the search for new antimicrobial drugs.

## Data Availability Statement

The original contributions presented in the study are included in the article/Supplementary Material, further inquiries can be directed to the corresponding author/s.

## Author Contributions

LP, EM, VM, MLB, MWB, and LS conducted the compound isolation, synthesis, and characterization. DC, LW, and SW conducted the antimicrobial activity and cytotoxicity work. DC, LW, SW, and LS wrote the manuscript. All authors contributed to the design of the experiments and reviewed the manuscript.

## Conflict of Interest

The authors declare that the research was conducted in the absence of any commercial or financial relationships that could be construed as a potential conflict of interest.

## Supplementary Material

Supplementary Figure 1 Dose response curves of *M. tuberculosis* treated with isoniazid or linezolid. *M. tuberculosis* was treated for 7 days with drug concentrations ranging from 200 μg/mL to 0.09 μg/mL, in three-fold serial dilutions. Bacterial survival was measured after overnight incubation with CellTiter-Blue to establish minimum inhibitory concentrations (MICs). Data points are expressed as mean % survival relative to drug-free controls from duplicate biological replicates. Error bars represent the standard deviation.
