# Supplementary figures and images for: Search for Antimicrobial Activity Among Fifty-Two Natural and Synthetic Compounds Identifies Anthraquinone and Polyacetylene Classes That Inhibit Mycobacterium tuberculosis
Source: Front Microbiol. 2021 Jan 18;11:622629. doi: 10.3389/fmicb.2020.622629 (PMC7847937; doi:10.3389/fmicb.2020.622629)

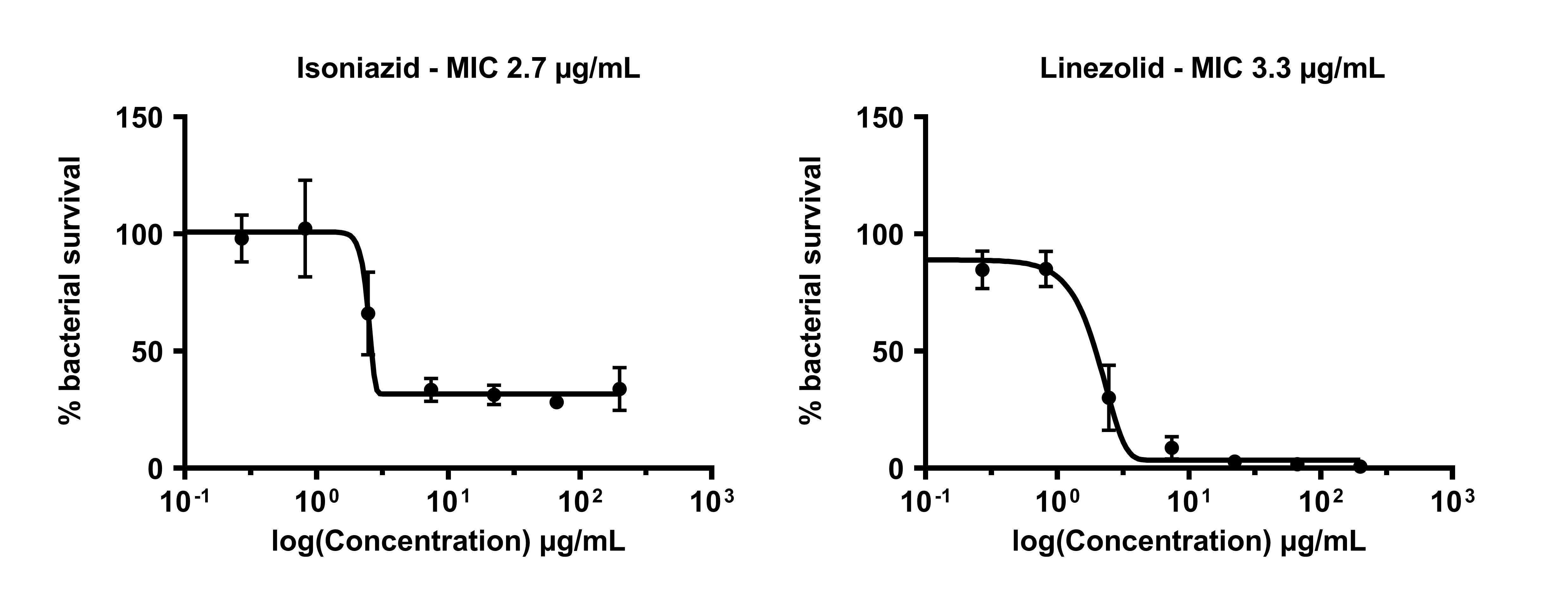

Supplement: Supplementary Figure 1 — Dose response curves of M. tuberculosis treated with isoniazid or linezolid. M. tuberculosis was treated for 7 days with drug concentrations ranging from 200 μg/mL to 0.09 μg/mL, in three-fold serial dilutions. Bacterial survival was measured after overnight incubation with CellTiter-Blue to establish minimum inhibitory concentrations (MICs). Data points are expressed as mean % survival relative to drug-free controls from duplicate biological replicates. Error bars represent the standard deviation. [file Image_1.TIF]
